# Experimental Study on Artificial Cemented Sand Prepared with Ordinary Portland Cement with Different Contents

**DOI:** 10.3390/ma8073960

**Published:** 2015-07-02

**Authors:** Dongliang Li, Xinrong Liu, Xianshan Liu

**Affiliations:** 1 Key Laboratory of New Technology for Construction of Cities in Mountain Area (Chongqing University), Ministry of Education, Chongqing 400045, China; E-Mails: liuxrong@126.com (X.L.); lzmoumou@163.com (X.L.); 2 School of Civil Engineering, Chongqing University, Chongqing 400045, China

**Keywords:** artificial, cemented sand, ordinary Portland cement, test

## Abstract

Artificial cemented sand test samples were prepared by using ordinary Portland cement (OPC) as the cementing agent. Through uniaxial compression tests and consolidated drained triaxial compression tests, the stress-strain curves of the artificial cemented sand with different cementing agent contents (0.01, 0.03, 0.05 and 0.08) under various confining pressures (0.00 MPa, 0.25 MPa, 0.50 MPa and 1.00 MPa) were obtained. Based on the test results, the effect of the cementing agent content (*C*_v_) on the physical and mechanical properties of the artificial cemented sand were analyzed and the Mohr-Coulomb strength theory was modified by using *C*_v_. The research reveals that when *C*_v_ is high (e.g., *C*_v_ = 0.03, 0.05 or 0.08), the stress-strain curves of the samples indicate a strain softening behavior; under the same confining pressure, as *C*_v_ increases, both the peak strength and residual strength of the samples show a significant increase. When *C*_v_ is low (e.g., *C*_v_ = 0.01), the stress-strain curves of the samples indicate strain hardening behavior. From the test data, a function of *C*_v_ (the cementing agent content) with *c*′ (the cohesion force of the sample) and Δϕ′ (the increment of the angle of shearing resistance) is obtained. Furthermore, through modification of the Mohr-Coulomb strength theory, the effect of cementing agent content on the strength of the cemented sand is demonstrated.

## 1. Introduction

Cemented sand is widely found in nature. It can be formed in multiple ways, such as precipitation of silicon dioxide and oxidation reaction of the ferric oxide in the sand [1,2,3,4]. There are three kinds of cements, which are classified, according to their compositions, as siliceous cement, carbonate cement and clay mineral cement. Since the 1970s, scholars have been researching cemented sand. Usluogullari *et al*. [5] studied the effect of curing time on the physical and mechanical properties of cemented sand through triaxial compression test on the artificial cemented sand sample produced with OPC. Trads *et al*. [6] conducted triaxial testing and discrete element method (DEM) analysis on cement bond sand and gypsum cement sand samples, and found that the mechanical properties of cemented sand depend not only on density and confining pressure of the sample, but also on the cement ratio. Thian *et al*. [7] examined the stress-strain behavior of the artificial cemented sand sample produced with ordinary Portland cement under low confining pressure. By comparing it with uncemented sand, they found that cemented sand has greater strength and stiffness. Coop *et al*. [8,9] carried out triaxial compression tests on calcareous sandstone, silica sandstone and artificial cemented sand, and stated that sand should be defined not only by its chemical composition, but by its structure during natural formation. Xumin Wang *et al*. [10] prepared calcium oxide cemented sand with a secondary water blending method, analyzed the influence of the added amount of calcium oxide on the physical and mechanical properties of cemented sand, and defined the chemical index β to manifest the yield of cement. Nardelli *et al*. [11] studied the mesoscopic mechanical properties of artificial cemented sand through experiments. Ravi *et al*. [12] modified silt soil by mixing a certain proportion of desulfurized fly ash and lime, and through triaxial tests reached the conclusion that conventional sand can be liquefied and mixed soil samples cannot, but they have improved cementation and strength. Asghari *et al*. [13] established a three-dimensional grain flow numerical model through mesoscopic mechanical analysis to simulate the shear properties of cemented sandstone, identified its mechanical parameters, and studied its failure mechanism. Rios *et al*. [14,15] analyzed the compression behavior of an artificially cemented soil with the adjusted porosity/cement index using a correlation. In order to evaluate the effects of cementation on the mechanical properties of cement-treated soil, a series of isotropic consolidation and undrained triaxial compression shear tests were performed by Kasama *et al*. [16]. Also, the stress-strain behavior and mechanical characterization [17,18,19,20,21,22], the influence of grain size and mineral composition of granular soils [23] are also researched.

By reviewing the references [24,25,26,27,28,29,30,31,32,33], it can be seen that current research on cemented sand is mainly concerned with the mechanical properties of the cemented sand samples and the establishment of a mechanical model. As we all know, the Mohr-Coulomb strength theory assumes the cohesion and the angle of shearing resistance to be constant values. However, for artificial cemented sand, its cohesion and the angle of shearing resistance vary with cementing agent content. This means the original Mohr-Coulomb strength theory expression is not suitable for artificial cemented sand with different cementing agent content. With this in mind, the artificial cemented sand test samples were prepared for this paper by using OPC with a strength grade of 42.5 as the cementing agent. Mechanical tests are conducted on these samples and the relationships between their physical and mechanical properties, mechanical indexes and the cementing agent content are analyzed. Based on the analysis, a modified Mohr-Coulomb strength theory expression is obtained.

## 2. Experimental

Taking the cemented sand sample readily available in nature can easily cause damage to its cementation, with the damage taking place in a random manner. As a result, it is difficult to get high quality cemented samples directly from nature. Additionally, the parameters of natural cemented sand are controlled by many factors. It is difficult for us to get natural cemented sand samples with same parameters. Therefore, artificial cemented sand is used for research in the test. Artificial cemented sand is an artificial sample made from sand, cementing agent and water, which is used for simulating the cementation state of sand in natural state.

### 2.1. Raw Materials

**Figure 1:**
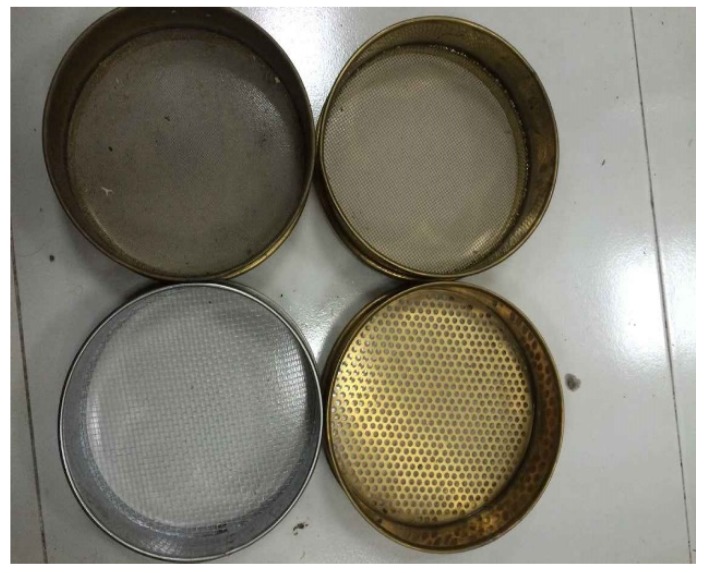
The circular sieves.

High-purity quartz sand (S_i_O_2_ > 99%) with grains of uniform diameter is used for the tests. To avoid severe adverse effects on the internal friction angle of the samples, spherical or elliptical sand grains should be selected. The larger irregular grains should be picked out directly. Then, several standard circular sieves (5 mm, 3.35 mm, 2 mm and 1 mm) were used successively for sand grain sieving. The circular sieves are shown in Figure 1.

The effect of the cementing agent content on the physical and mechanical properties of the cemented sand should not be overlooked. By virtue of its excellent cementing power, OPC (with a strength grade of 42.5) is used as the cementing agent in the test. In order to eliminate the effect of impurities in water, distilled water is used in the test to ensure accuracy of the test results.

### 2.2. Sample Preparation

**Figure 2:**
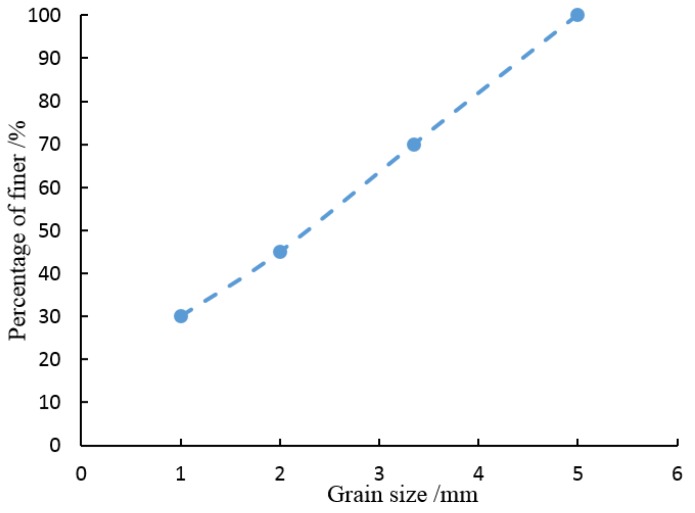
Grading curve for the screened quartz grains.

The standard circular sieves were used for sand grain sieving. The diameters of the sieves are 1 mm, 2 mm, 3.35 mm and 5 mm, respectively, as shown in Figure 1. The grading curve for the sieved quartz grains is shown in Figure 2. The sieved sand particles were thoroughly washed and oven dried at 100 °C for 24 h before use. The parameters of quartz sand are shown in Table 1. The relative density *D*_r_ is calculated from *e*, *e*_min_ and *e*_max_. The formula is as follows:

(1) $$D_{\text{r}}=\frac{e_{\text{max}}-e}{e_{\text{max}}-e_{\text{min}}}$$

where *D*_r_ is the relative density, *e* is the real void ratio of the quartz sand, *e*_max_ is the maximum void ratio of the quartz sand, *e*_min_ is the minimum void ratio of the quartz sand. When *D*_r_ = 0, *e* = *e*_max_, this means that the sand is in the loosest state. When *D*_r_ = 0, *e* = *e*_max_, which this that the sand is in the most dense state.

**Table 1:** The parameters of the quartz sand.

Relative Density *D*_r_	Dry Density *ρ*_d_/(g/cm^3^)	Void Ratio *e*
1.0	1.77	0.50
0.8	1.58	0.62
0.0	1.11	0.70

**Figure 3:**
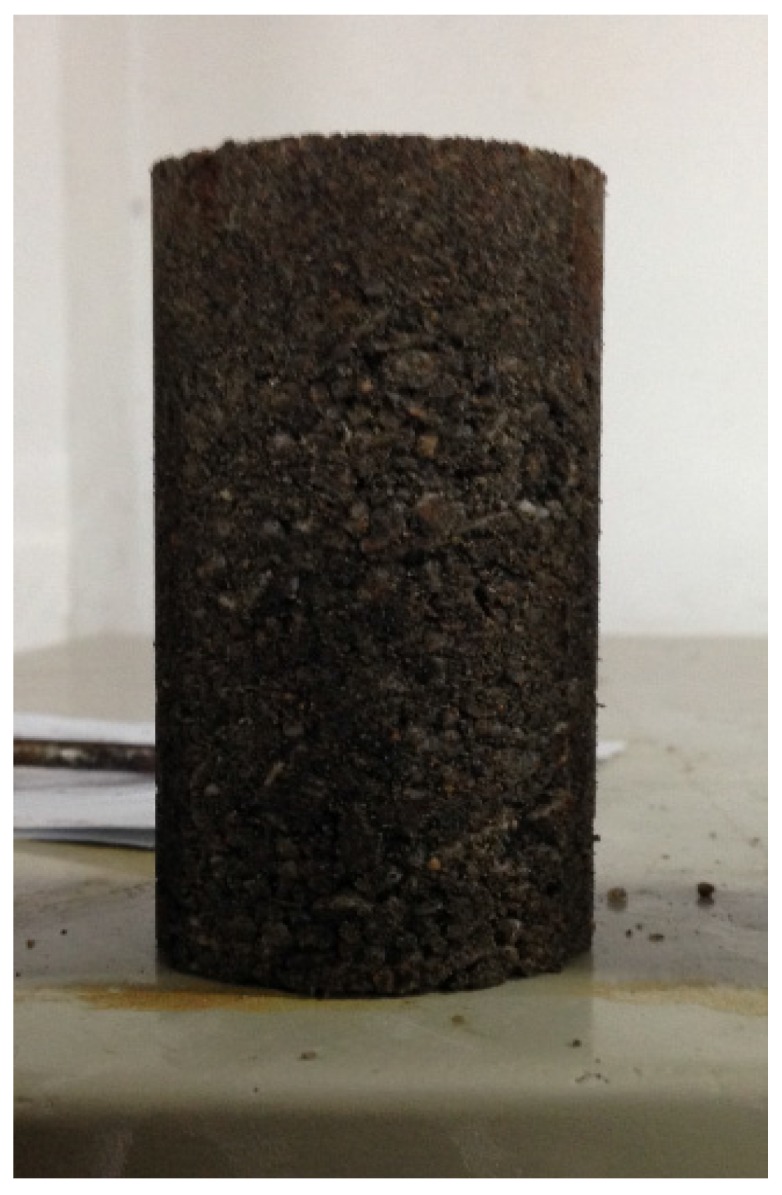
Sample of artificial cemented sand.

The quartz sand should be mixed well with different proportions of OPC such that the OPC grains are evenly distributed over the surface of the sand grains. After mixing, water was added at a cementing agent-water ratio of 1:1 by mass and stirred. Then the mixed material was filled into the detachable cylindrical sample mould. To ensure uniformity of the sample, the mixed material was poured into the mould in 8 layers and compacted layer by layer. The sample had a diameter of 50 mm and a height of 100 mm, as shown in Figure 3. Four kinds of artificial cemented sand samples were prepared with OPC content *C_v_* (mass of OPC/mass of quartz sand) being 0.01, 0.03, 0.05 and 0.08 respectively, so as to facilitate the study on the effect of cementing agent content on the physical and mechanical properties of artificial cemented sand. The compacted specimens were sealed in a plastic bag under a constant temperature of 25 °C to minimize moisture loss during the curing process and cracking caused by temperature variations. The curing time for artificial cemented sand prepared with OPC was 28 days. See Table 2 for parameters of the quartz sand.

**Table 2:** Parameters of cemented sand samples.

Relative Density *D*_r_	Dry Density *ρ*_d_/(g/cm^3^)	Dry Mass (g)	Mass of Quartz Sand (g)	*C*_v_	Mass of Cementing Agent (g)	Mass of Water (g)
0.8	1.58	310	306.9	0.01	3.1	3.1
			301.0	0.03	9.0	9.0
			295.2	0.05	14.8	14.8
			287.0	0.08	23.0	23.0

### 2.3. Testing Method

**Figure 4:**
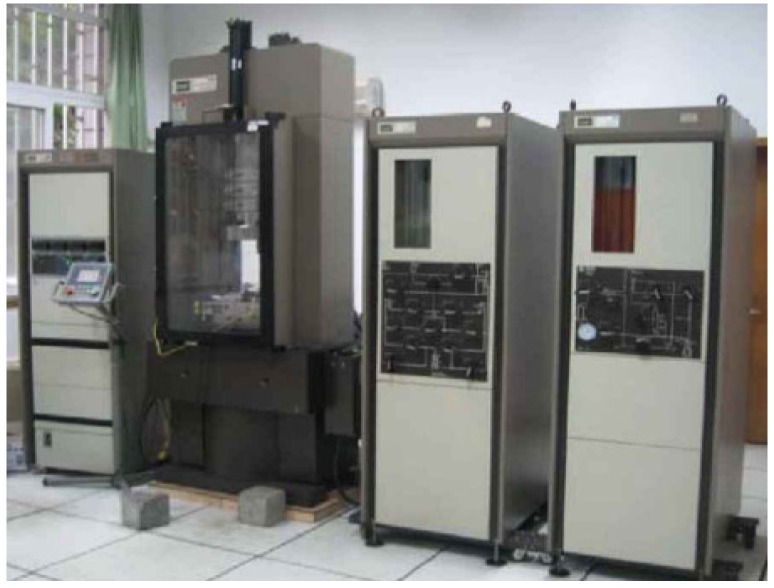
MTS 815 Rock Material Tester.

The drained triaxial compression tests were performed using an MTS 815 Rock Material tester (MTS Industrial Systems Company, Eden Prairie, MN, USA), which is as shown in Figure 4.

**Figure 5:**
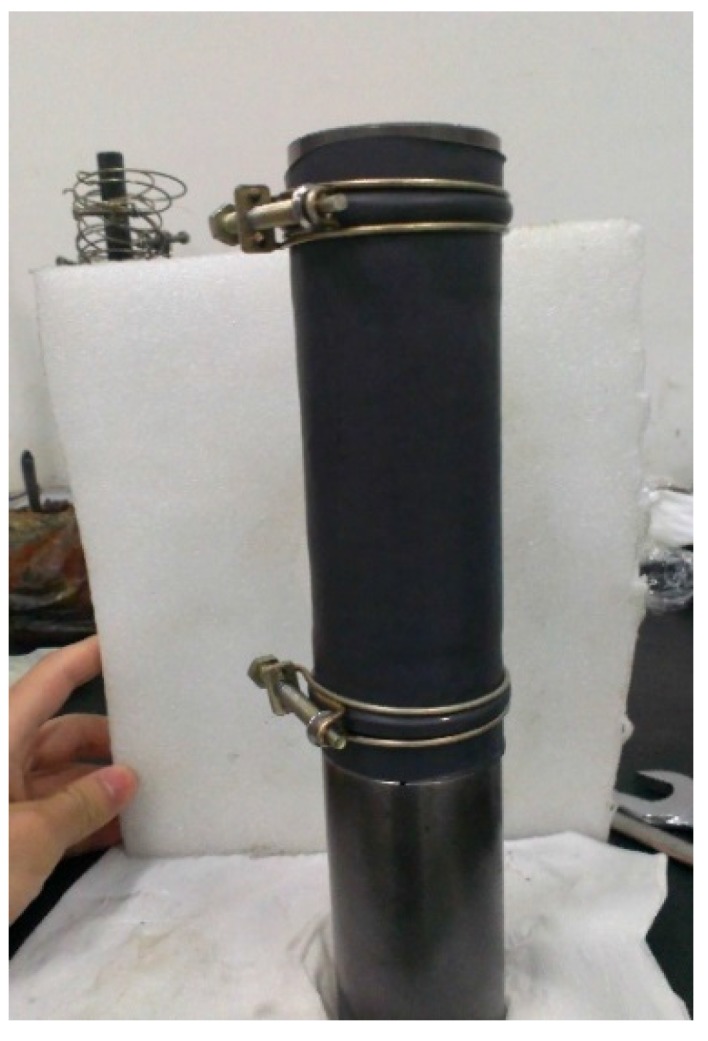
Covering the sample with a rubber sleeve.

Deaired water was circulated to saturate the specimens. For higher accuracy, 3 parallel samples were used in each test group. Triaxial compression tests under various confining pressures (0.25 MPa, 0.50 MPa and 1.00 MPa) were performed on the artificial cemented sand samples prepared with OPC of different contents (0.01, 0.03, 0.05 and 0.08). According to Wang *et al*. [34], the shearing was carried out with an axial-strain rate of 6%/h. To prevent damage to the tester by the sand grains falling from a damaged sample, the sample was covered with a rubber sleeve and fastened securely, as shown in Figure 5.

**Figure 6:**
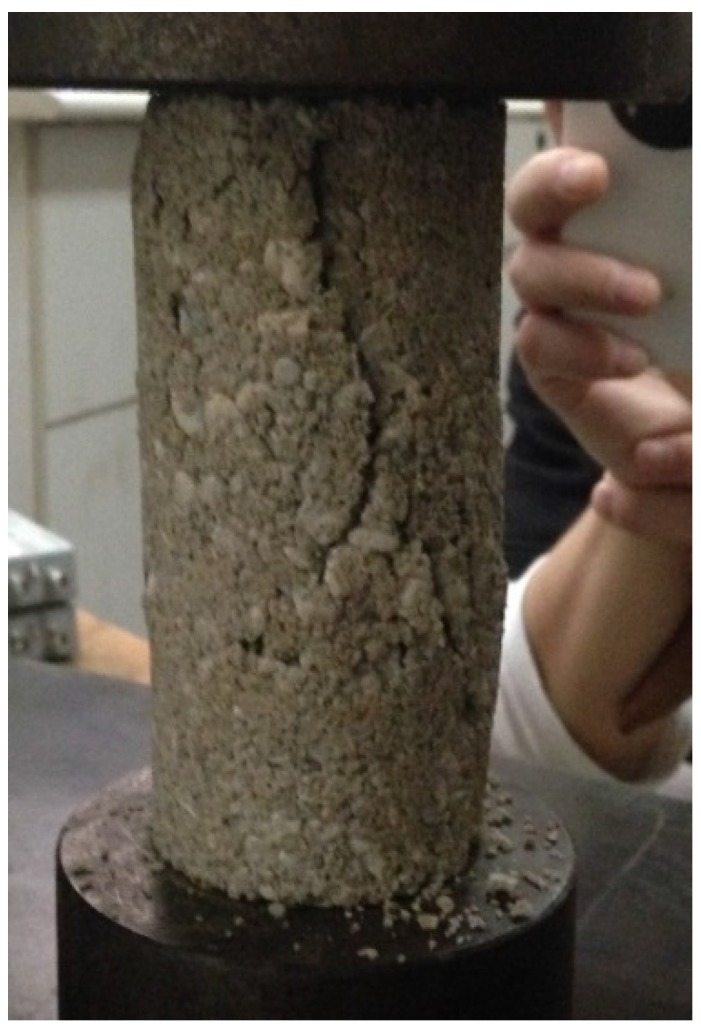
Uniaxial compression test on the samples.

To obtain the stress-strain curve of artificial cemented sand samples prepared with OPC under a confining pressure of 0 MPa, uniaxial compression test on the samples was required (see Figure 6). 

## 3. Results and Discussion

### 3.1. Stress-Strain Curve

**Figure 7:**
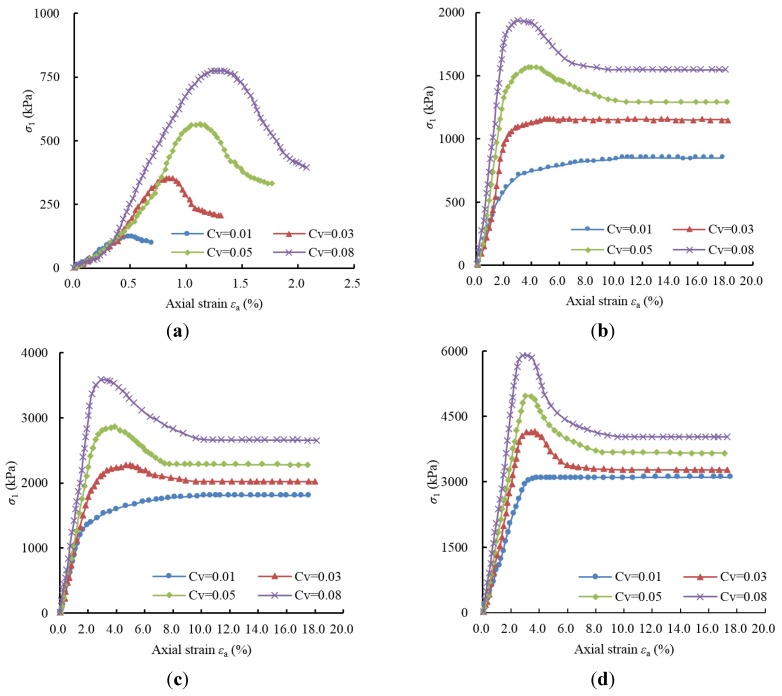
Stress-strain relationships of OPC cement sand samples under different conditions: (**a**) 0.00 MPa; (**b**) 0.25 MPa; (**c**) 0.50 MPa; (**d**) 1.00 MPa.

Figure 7 provides the principal stress (σ_1_)-axial strain (ε_α_) relationship curve obtained from the drained triaxial compression test under various confining pressures (0.00 MPa, 0.25 MPa, 0.50 MPa and 1.00 MPa) on the artificial cemented sand prepared with OPC of different contents (0.01, 0.03, 0.05 and 0.08).

For artificial cemented sand samples with higher cementing agent content (0.03, 0.05 and 0.08), it can be seen in Figure 7 that under the same confining pressure, the increase in the content of OPC leads to a remarkable increase in peak strength (principal stress σ_1_) of the sample. For instance, when the confining pressure is 0.25 Mpa, the peak strength of the sample with *C*_v_ = 0.03 is 1155.7 kPa; when *C*_v_ increases to 0.05, its peak strength is 1573.2 kPa, an increase of 417.5 kPa; when *C*_v_ increases to 0.08, its peak strength is 1937.1 kPa, another increase of 363.9 kPa. Apart from the peak strength, the residual strength of the samples increases with *C*_v_ as well. Moreover, as can be seen from the figure, the peak strength of the samples is sharper, and their strain softening behavior is more apparent. In general, the σ_1_-ε_α_ curve consists of the following stages: initial stress rise → stress rise getting slower→ peak stress → plastic softening → residual strength. The addition of OPC drives cementation between sand grains and effectively enhances the strength and integrity of the quartz sand. Therefore, more cementing agent can significantly improve the strength of artificial cemented sand and change its stress-strain properties. This is consistent with the results achieved by Schnaid [35] and Ismail *et al*. [36]. 

For artificial cemented sand samples with lower cementing agent content (0.01), it can be seen in Figure 7b–d that the stress-strain curves show no obvious peak point and demonstrate the strain hardening behavior of the samples instead. This is radically different from the samples with higher cementing agent content (0.03, 0.05 and 0.08). The reason is that low cementing agent content is not sufficient to fully fill the spaces between the sand grains, and still less to cover the surface of the sand grains. This causes the bond between sand grains to be inadequate and weak. In this case, if a confining pressure is applied, the bond between the sand grains is gradually destroyed, the artificial cemented sand is transformed to unconsolidated quartz sand, and volume of the sample is compressed. Meanwhile, the existence of confining pressure drives the generation of bite force between sand grains, which increases the stress. This phenomenon is also known as strain hardening.

### 3.2. Relationship between Shear Strength Index and Cementing Agent Content

The Mohr-Coulomb strength theory is one of the strength theories widely acknowledged by scholars around the world. It is expressed as follows:

(2) $${\text{τ}}_{\text{f}}=c^{\prime }+{\text{σ}}^{\prime }\text{tan}\phi^{\prime }$$

where τ_f_ is the shear strength (MPa), *c*′ is the cohesion force (MPa), σ′ is the effective confining pressure (MPa) and ϕ′ is the angle of shearing resistance (°). 

It can be easily seen that τ_f_ is determined by *c*′, σ′ and ϕ′. In this paper, the strength theory is employed for strength index fitting, the results of which are used to extract the cohesion *c*′ and the angle of shearing resistance ϕ′ for analysis. Table 3 shows the peak stress of artificial cemented sand samples with different contents of OPC under various confining pressures in the test.

**Figure 8:**
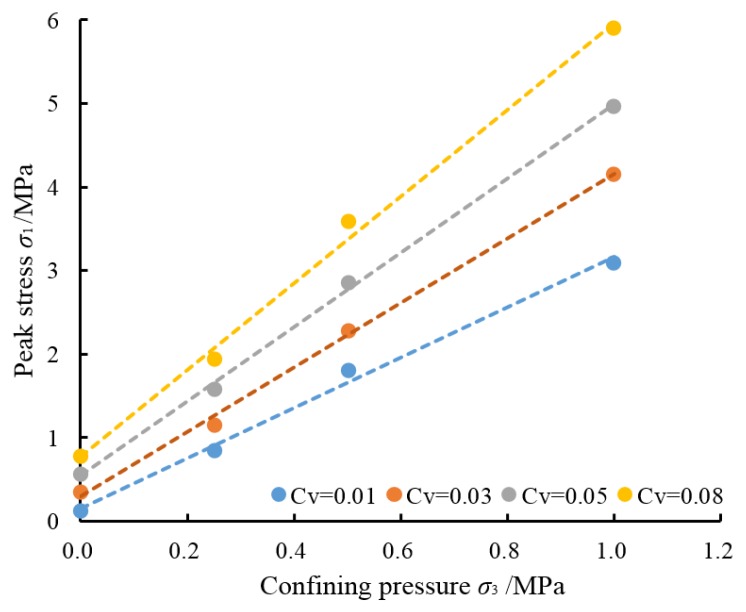
The peak stress of samples under different conditions.

According to Table 3, a diagram can be drawn to show the relationship between the peak stress of samples with different cementing agent content and the confining pressure. See Figure 8. 

The Mohr-Coulomb criterion represented by the principal stress can be expressed as [37]

(3) $${\text{σ}}_{1}=A{\text{σ}}_{3}+B$$

where $A=\frac{1+\text{sin}\phi^{\prime }}{1-\text{sin}\phi^{\prime }}$ and $B=\frac{2c^{\prime }\text{cos}\phi^{\prime }}{1-\text{sin}\phi^{\prime }}$.

(4) $$\phi^{\prime }=acr\text{sin}\frac{A-1}{A+1}$$

(5) $$c^{\prime }=\frac{B(1-\text{sin}\phi^{\prime })}{2\text{cos}\phi^{\prime }}$$

As can be seen from the figure above, the strength of the artificial cemented sand prepared with OPC increases as the confining pressure get higher and there is an approximately linear relationship between them, which is largely in line with the Mohr-Coulomb strength criterion. 

Through regression analysis on relevant data in Table 3, the formulas of the regression curves are obtained, as shown in Table 4. According to Equation (3) and the formulas in Table 4, parameters A and B can be obtained. With Equations (4) and (5), the calculated values of *c*′ and ϕ′ of the artificial cemented sand prepared with OPC can be obtained, as shown in Table 4. The correlation coefficient *R*^2^ are all greater than 0.99, which means that the values of *c*′ and ϕ′ are comparatively accurate.

**Table 3:** The peak stress of samples under different conditions.

*C*_v_	Confining Pressure (MPa)	Peak Stress σ_1_ (MPa)
0.01	0.00	0.1232
	0.25	0.8518
	0.50	1.8123
	1.00	3.1034
0.03	0.00	0.3520
	0.25	1.1557
	0.50	2.2732
	1.00	4.1578
0.05	0.00	0.5643
	0.25	1.5732
	0.50	2.8576
	1.00	4.9673
0.08	0.00	0.7752
	0.25	1.9371
	0.50	3.5839
	1.00	5.8936

**Table 4:** Values of *R^2^*, *c*′ and ϕ′*.*

*C*_v_	Formula	Correlation Coefficient *R^2^*	Cohesion Force *c*′ (MPa)	Angle of Shearing Resistance ϕ′(°)
0.01	*σ*_1_ = 3.0086*σ*_3_ + 0.1564	0.9938	0.0451	30.07
0.03	*σ*_1_ = 3.8585*σ*_3_ + 0.2966	0.9980	0.0754	36.04
0.05	*σ*_1_ = 4.4450*σ*_3_ + 0.9459	0.9985	0.1295	39.39
0.08	*σ*_1_ = 0.1873*σ*_3_ + 0.7780	0.9953	0.1708	42.59

**Figure 9:**
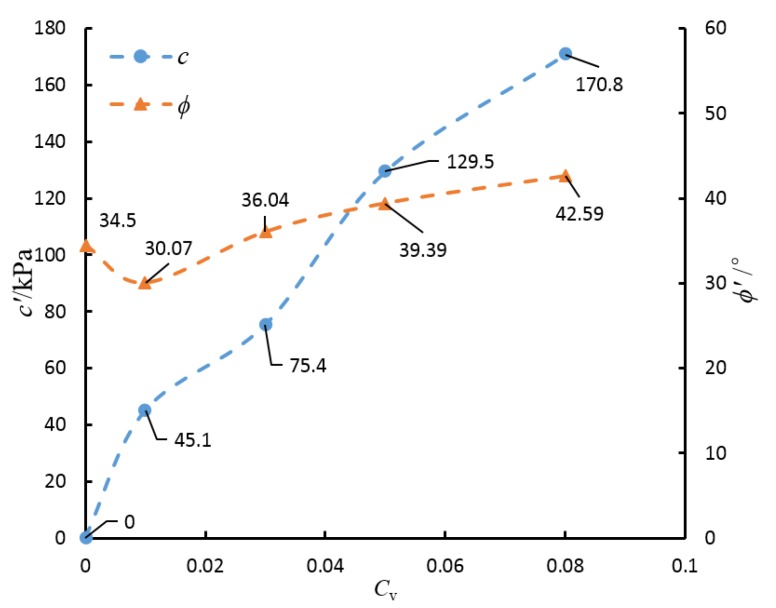
The relationship curves for *C*_v_ and *c*′ (ϕ′)

Through tests, it was shown that the internal friction angle of the quartz sand used is 34.5°. By combining this with the data in Table 4, the curves that denote the relations between *C*_v_ and *c*′ (ϕ*′*) are acquired, as shown in Figure 9. As the quartz sand itself is cohesionless, its cohesion force is 0. With the addition of OPC, the quartz grains are cemented and *c*′ becomes a non-zero value. As shown in Figure 9, the increase of *C*_v_ results in a significant increase in the cohesion force *c*′ of the artificial cemented sand. When *C*_v_ = 0.01, the cohesion force *c*′ of the sample increases by 45.1 KPa, compared with the quartz sand; when *C*_v_ = 0.03, *c*′ increases by 30.3 kPa from *C*_v_ = 0.01; when *C*_v_ = 0.05, *c*′ increases by 54.1 kPa from *C*_v_ = 0.03; when *C*_v_ = 0.08, *c*′ increases by 41.3 kPa from *C*_v_ = 0.05. As the quartz sand is cohensionless, the value of *c*′ in this paper is used to represent the cementation of OPC between quartz grains. Moreover, it can be seen from the figure that the angle of shearing resistance of the sample ϕ′ is 30.07° when *C*_v_ = 0.01, smaller than that of the quartz sand. It is probably due to the addition of cohesion to the strength criterion. As for quartz sand, *c*′ = 0. With the increasing of *C*_v_, *c*′ of the cemented sand increases. If the cemented data was fitted with a strength envelope with no cohesion, the angle of shearing resistance was certainly higher than 34.5.With the continuing increase of *C*_v_, ϕ′ increases as well, but at a slower pace. When *C*_v_ = 0.03, the angle of shearing resistance ϕ′ of the sample shows an increase of 5.97° as compared with *C*_v_ = 0.01; when *C*_v_ = 0.05, ϕ′ shows an increase of 3.35° as compared with *C*_v_ = 0.03; when *C*_v_ = 0.08, ϕ′ shows an increase of 3.20° as compared with *C*_v_ = 0.05.

Δ*c* (MPa) is defined as the change of *c*′ for the artificial cemented sand with *C*_v_, Δϕ′ (°) as the change of ϕ′ with *C*_v_, while *i*_c′_ (%) and *i*_ϕ′_ (%) are the change rates corresponding to *c*′ and ϕ*′* respectively. Based on the values of *c*′ and ϕ′ when *C*_v_ is 0.01, the change in *c*′ and ϕ′ with the cementing agent content is shown in Table 5. 

**Table 5:** The change of *c*′ and *ϕ*′ with different *C*_v_.

C_v_	Δc′ (MPa)	*i*_c__′_ (%)	Δϕ′ (°)	*i*_ϕ__′_ (%)
0.03	0.0303	67.18	5.97	19.85
0.05	0.0844	187.14	9.32	30.99
0.08	0.1257	278.71	12.52	41.64

According to the Table 5, the values of ϕ′ and *c*′ obviously increase with *C*_v_ and the growth rates are high. With *i*_c′_ and *i*_ϕ′_ at *C*_v_ = 0.01 excluded, the average increase rate of *c*′ is 39.81%, and that of ϕ′ is 5.95%. Considering the typical range of the angle of shear resistance, ϕ′ increased from 30.07° to 42.59°, which is quite significant (41.64%). 

**Figure 10:**
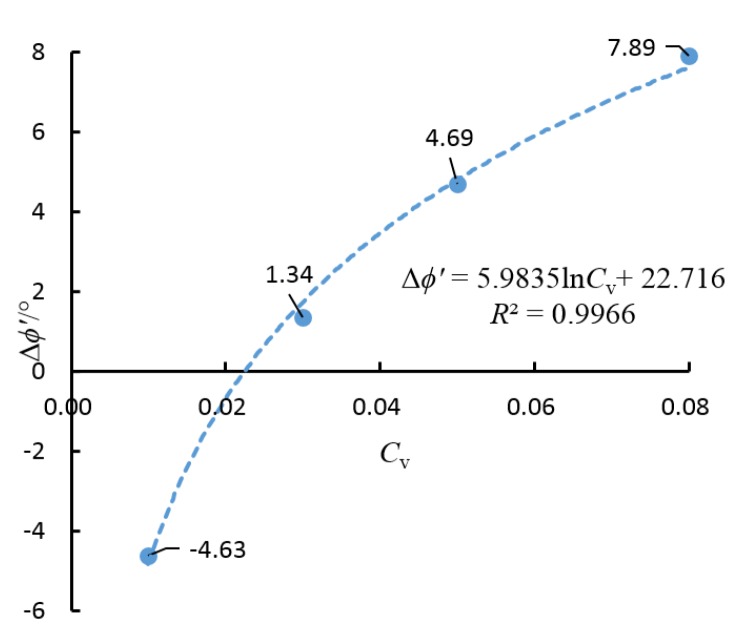
The relationship curve for Δϕ′ and *C*_v_.

**Figure 11:**
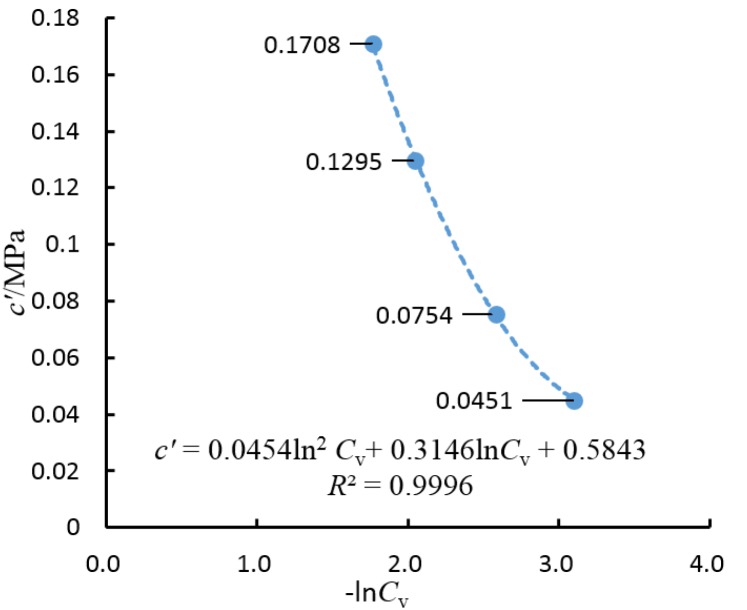
The relationship curve for *c*′ and −ln*C*_v_.

The artificial cemented sand prepared with OPC has a cohesion force (*c*′) and an internal friction angle (ϕ′) that change with *C*_v_. Δϕ′ is defined as the increment of the angle of shearing resistance ϕ′. The relationship curve about Δϕ′ and *C*_v_ is shown in Figure 10 and the relationship curve for *c*′ and −ln*C*_v_ is shown in Figure 11. 

Δϕ′ and *C*_v_ are fitted with a log function, while *c*′ and −ln*C*_v_ are fitted with a quadratic polynomial function. According to Figure 10 and Figure 11, it is known that

(6) $$\Delta \phi^{\prime }=a\text{ln}C_{v}+b$$

(7) $$c^{\prime }=m{\text{ln}}^{2}C_{v}+nC_{v}+k$$

where *a*, *b*, *m*, *n* and *k* are parameters. 

For the artificial cemented sand samples prepared using OPC as the cementing agent, through curve fitting based on the test data and Equations (6) and (7), the following can be obtained: 

(8) $$\Delta \phi^{\prime }=5.9835\text{ln}C_{v}+22.716$$

(9) $$c^{\prime }=0.0454{\text{ln}}^{2}C_{v}+0.3146C_{v}+0.5843$$

The correlation coefficients of the fitted curves are 0.9966 and 0.9996, respectively. Therefore, the assumptions that Δϕ′ and *C*_v_ follow a logarithmic equation and *c*′ and −ln*C*_v_ follow a quadratic polynomial equation are rational. 

The Mohr-Coulomb strength theory assumes the angle of shearing resistance ϕ′ to be a constant, that is, the strength envelope is a straight line. However, for artificial cemented sand, its cohesion force *c*′ and the angle of shearing resistance ϕ′ vary with *C*_v_. Based on analysis of the test results and the cementing agent content *C*_v_, a modified Mohr-Coulomb strength theory expression is obtained by substituting Equations (6) and (7) into Equation (2):

(10) $${\text{τ}}_{\text{f}}=m{\text{ln}}^{2}C_{v}+nC_{v}+k+{\text{σ}}^{\prime }\text{tan}(\phi^{\prime }+a\text{ln}C_{v}+b)$$

where *τ*_f_ is the shear strength (MPa), *c*′ is the cohesion force (MPa), σ′ is the confining pressure (MPa), ϕ′ is the angle of shearing resistance (°) of the sand grains, *C*_v_ is the cementing agent content, and *a*, *b*, *m*, *n* and *k* are parameters. 

In addition, when *C*_v_ = 0, Equation (10) is reduced to: 

(11) $${\text{τ}}_{\text{f}}={\text{σ}}^{\prime }\text{tan}\phi^{\prime }$$

For the artificial cemented sand prepared with OPC in this test, its *C*_v_-related Mohr-Coulomb strength theory expression is as follows: 

(12) $${\text{τ}}_{\text{f}}=0.0454{\text{ln}}^{2}C_{v}+0.3146C_{v}+0.5843+{\text{σ}}^{\prime }\text{tan}(\phi^{\prime }+5.9835\text{ln}C_{v}+22.716)$$

## 4. Conclusions

In this study, OPC with a strength grade of 42.5 was used as the cementing agent. Different proportions of OPC were mixed with high-purity quartz sand and distilled water to prepare the artificial cemented sand samples. These samples, after 28 days of curing, were subjected to uniaxial compression and triaxial compression tests under various levels of confining pressure. Based on the stress-strain curves obtained from the tests and test data analysis and fitting, the following conclusions can be drawn:

(1) The added amount of OPC *C*_v_ has an effect on the physical and mechanical properties of artificial cemented sand. When *C*_v_ is high (e.g., *C*_v_ = 0.03, 0.05 or 0.08), the stress-strain curve indicates a strain softening behavior of the samples; under the same confining pressure, as *C*_v_ increases, both the peak strength and residual strength of the samples show a significant increase. When *C*_v_ is low (e.g., *C*_v_ = 0.01), the stress-strain curve indicates the strain hardening behavior of the samples.
(2) With the increase of *C*_v_, the cohesion force *c*′ and Δϕ′ gradually increase. The angle of shearing resistance of the artificial cemented sand is smaller than that of the uncemented sample when *C*_v_ is low; as *C*_v_ increases, it increases as well, but in a moderate manner. *c*′ is much more sensitive than ϕ′ to the change in the cementing agent content.
(3) For the artificial cemented sand prepared with OPC, Δϕ′ and *C*_v_ conform to the rule of logarithmic function, while *c*′ and −ln*C*_v_ conform to the rule of quadratic polynomial function. The correlation coefficients of both functions are greater than 0.9965, indicating an extremely strong correlation.
(4) Given the effect of *C*_v_ on the shear strength of the artificial cemented sand, the Mohr-Coulomb strength theory was modified and an expression based on this theory appropriate for artificial cemented sand with different levels of cementing agent content is presented.
